# Hexavalent chromium induces malignant transformation of human lung bronchial epithelial cells via ROS-dependent activation of miR-21-PDCD4 signaling

**DOI:** 10.18632/oncotarget.9967

**Published:** 2016-06-13

**Authors:** Poyil Pratheeshkumar, Young-Ok Son, Sasidharan Padmaja Divya, Lilia Turcios, Ram Vinod Roy, John Andrew Hitron, Lei Wang, Donghern Kim, Jin Dai, Padmaja Asha, Zhuo Zhang, Xianglin Shi

**Affiliations:** ^1^ Center for Research on Environmental Disease, University of Kentucky, Lexington, KY, USA; ^2^ Department of Toxicology and Cancer Biology, University of Kentucky, Lexington, KY, USA; ^3^ Department of Surgery, University of Kentucky, College of Medicine, Lexington, KY, USA; ^4^ National Centre for Aquatic Animal Health, Cochin University of Science and Technology, Cochin, India

**Keywords:** hexavalent chromium, ROS, miR-21-PDCD4 signaling, IL-6, STAT3

## Abstract

Hexavalent chromium [Cr(VI)] is a well-known human carcinogen associated with an increased risk of lung cancer. However, the mechanisms underlying Cr(VI)-induced carcinogenesis remain unclear. MicroRNA-21 (miR-21) is a key regulator of oncogenic processes. Studies have shown that miR-21 exerts its oncogenic activity by targeting the tumor suppressor gene programmed cell death 4 (PDCD4). The present study examined the role of miR-21-PDCD4 signaling in Cr(VI)-induced cell transformation and tumorigenesis. Results showed that Cr(VI) induces ROS generation in human bronchial epithelial (BEAS-2B) cells. Chronic exposure to Cr(VI) is able to cause malignant transformation in BEAS-2B cells. Cr(VI) caused a significant increase of miR-21 expression associated with an inhibition of PDCD4 expression. Notably, STAT3 transcriptional activation by IL-6 is crucial for the Cr(VI)-induced miR-21 elevation. Stable knockdown of miR-21 or overexpression of PDCD4 in BEAS-2B cells significantly reduced the Cr(VI)-induced cell transformation. Furthermore, the Cr(VI) induced inhibition of PDCD4 suppressed downstream E-cadherin protein expression, but promoted β-catenin/TCF-dependent transcription of uPAR and c-Myc. We also found an increased miR-21 level and decreased PDCD4 expression in xenograft tumors generated with chronic Cr(VI)-exposed BEAS-2B cells. In addition, stable knockdown of miR-21 and overexpression of PDCD4 reduced the tumorogenicity of chronic Cr(VI)-exposed BEAS-2B cells in nude mice. Taken together, these results demonstrate that the miR-21-PDCD4 signaling axis plays an important role in Cr(VI)-induced carcinogenesis.

## INTRODUCTION

Hexavalent chromium compounds [Cr(VI) compounds] have been classified as human carcinogens by the International Agency for Research on Cancer (IARC) of the World Health Organization (WHO) based on epidemiological studies results linking Cr(VI) to lung cancer [1]. Cr(VI) compounds are widely used in industries, such as plating, paint, steel, tanning, and chrome ore processing [2]. It has been reported that overproduction of reactive oxygen species (ROS) play a major role in Cr(VI)-induced carcinogenesis [3]. However, a detailed molecular mechanism of Cr(VI)–induced malignant transformation and tumorigenesis remains unknown.

MicroRNAs (miRNAs) are a novel class of endogenous, small, noncoding RNAs that negatively regulate approximately 30% of the gene expression by a site-specific interactions at the 3′-UTR of target-mRNAs, causing translational repression or degradation [4, 5]. Among them, miR-21 has emerged as a key onco-miR [6], since it is consistently overexpressed in a number of human malignancies [7–10]. Increased expression of miR-21 has been associated with a variety of processes including in carcinogenesis, apoptosis resistance, cell proliferation, tumor progression and chemoresistance [11–14]. A recent study suggests that NADPH oxidase-derived ROS is essential for the expression and function of miR-21 [15].

PDCD4 is a novel tumor suppressor reported to inhibit neoplastic transformation, tumor promotion and progression [4, 16, 17]. It has been established that miR-21 directly targets the 3′ UTR region of PDCD4 and down-regulates its expression [18–20]. Moreover, high miR-21 levels were found to be inversely correlated with PDCD4 expression in a variety of tumors [18–24]. PDCD4 knockdown stimulated the invasion of colon cancer HT29 cells and inhibited E-cadherin expression. The reduced levels of E-cadherin elicited an accumulation of active β-catenin in the nuclei with a reported stimulation of β-catenin/T cell factor (TCF)-dependent transcription of genes such as uPAR and c-Myc [25, 26].

Persistent activation of signal transducer and activator of transcription-3 (STAT3) is frequently associated with malignant transformation [27]. Recent studies implicate STAT3 as a vital regulator of microRNA (miRNA) expression, and in addition, the STAT3 signaling pathway is controlled by several specific miRNAs [28–31]. There are two phylogenetically conserved STAT3 binding sites in miR-21 that regulate its oncogenic activity [30]. It has been reported that transcriptional activation of miR-21 by STAT3 leads to the induction of a stable transformed state in cancer cell lines [28]. Members of the IL-6 cytokine family are involved in a variety of biological responses, and the autocrine secretion of IL-6 contributes to cellular transformation via STAT3 phosphorylation [32].

In the present study, we investigated the interactive role of miR-21-PDCD4 signaling in Cr(VI)-induced malignant cell transformation. We found that chronic Cr(VI) exposure increased miR-21 levels and was associated with inhibition of PDCD4 expression and malignant cell transformation. Importantly, Cr(VI)-induced ROS was essential for the miR-21 elevation and PDCD4 reduction. In addition, STAT3 transcriptional activation by IL-6 was crucial for the Cr(VI)-induced miR-21 elevation. Furthermore, PDCD4 reduction by Cr(VI) suppressed downstream protein E-cadherin expression, and led to the β-catenin/TCF-dependent transcription of uPAR and c-Myc. These results suggest that miR-21 and PDCD4 are critical components of Cr(VI)-induced malignant transformation.

## RESULTS

### Cr(VI) increases miR-21 and targets PDCD4

**Figure 1:**
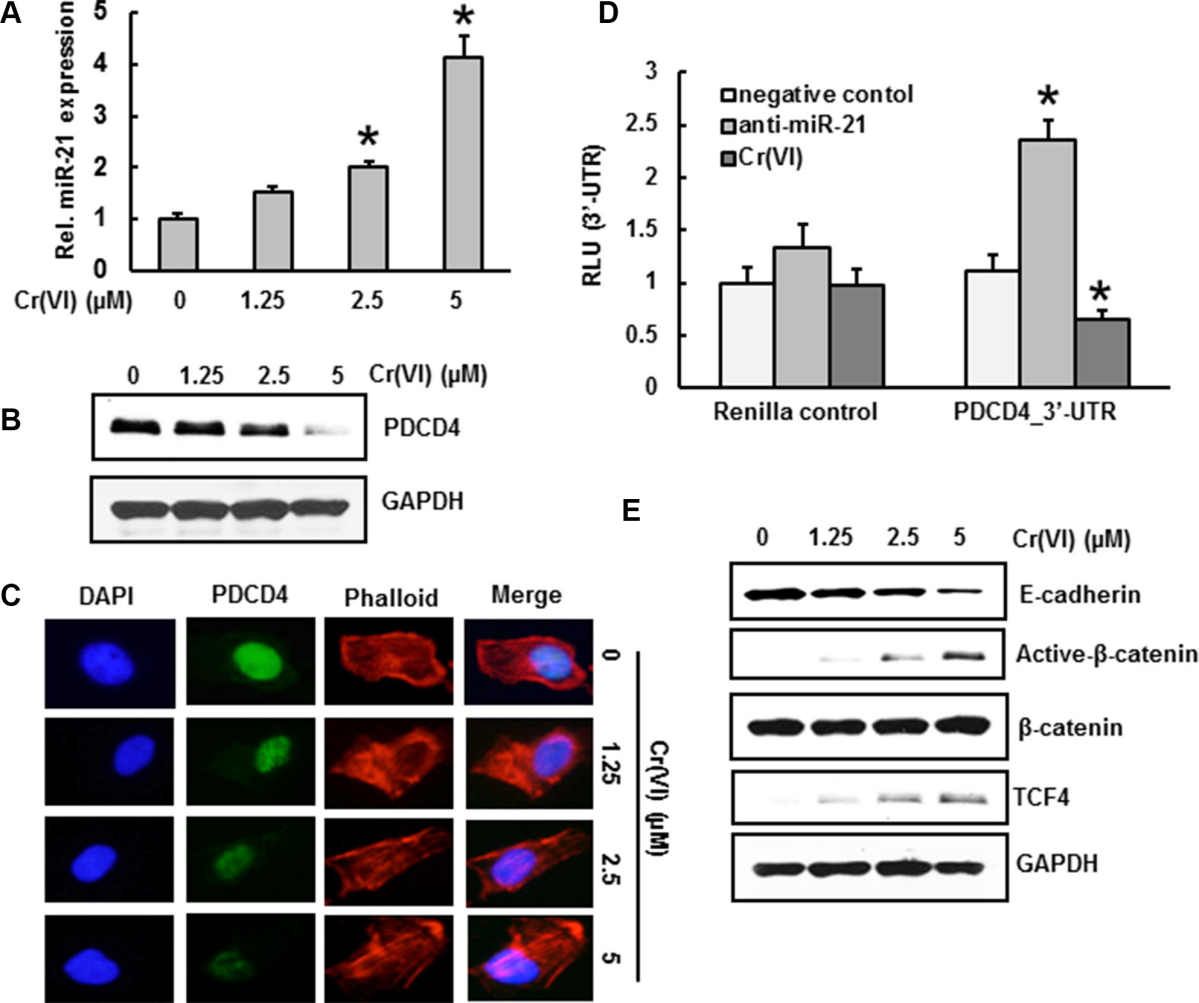
Cr(VI) increases miR-21 and targets PDCD4 BEAS-2B cells were exposed to increasing concentrations (0–5 μm) of Cr(VI) for 24 h. (**A**) The relative miR-21 level was determined by Taqman real-time PCR. (**B**) Immunoblot analysis of PDCD4 protein levels after acute Cr(VI) treatment. (**C**) Representative images of fluorescence immunostaining of PDCD4 (**D**) Cr(VI) increases the binding of miR-21 to the 3′-UTR of PDCD4. BEAS-2B cells were transfected with renilla reporter construct (pGL3-PDCD4_3′-UTR), miR-21 inhibitor (100 nM), negative control (100 nM), and pGL3-promoters and treated with 5 μM Cr(VI) for 6 h. Cellular lysates were subjected to a luciferase reporter analysis as described in Materials and Methods. The results are expressed as a relative activity (relative luminescence units (RLU)) normalized to the luciferase activity in the vector control cells without treatment. (**E**) Immunoblot analysis demonstrates that acute treatment of Cr(VI) decreases E-cadherin levels associated with an increase in β-catenin and TCF4 protein levels in BEAS-2B cells. Data presented in the bar graphs are the mean ± SD of three independent experiments. *indicates a statistically significant difference from control cells with *p* < 0.05.

Earlier studies showed that PDCD4, a novel tumor suppressor is an important functional target of the oncogenic microRNA miR-21 [33]. In this study we first examined whether acute treatment with Cr(VI) can increase miR-21 expression and downregulate its target tumor suppressor protein PDCD4. We observed a dose-dependent and significant (*p* < 0.05) elevation in the miR-21 levels associated with a dose-dependent decrease in PDCD4 expression by RT-PCR and Western blot analysis respectively in human bronchial epithelial BEAS-2B cells treated with Cr(VI) (Figure 1A and 1B). Similar results were observed by immunofluorescence analysis of PDCD4, where acute treatment of Cr(VI) diminished the PDCD4 expression in the nucleus (Figure 1C). There was a significant decrease in the PDCD4 3′-UTR reporter activity when cells were treated with 5 μM Cr(VI) for 6 h, whereas reporter activity was upregulated when miR-21 gene expression was inhibited (Figure 1D). These results support the assumption that acute Cr(VI) treatment increases the miR-21 levels with an associated decrease in PDCD4 expression.

### Cr(VI) regulates the downstream targets of PDCD4 -E-Cadherin, β-catenin and TCF4

Previous studies demonstrated that knock-down of PDCD4 down-regulates E-cadherin and increases β-catenin and TCF4 protein expression [26]. In the current study, acute treatment of BEAS-2B cells with Cr(VI) down-regulated E-cadherin protein expression with an associated up-regulation of active β-catenin (nuclear translocated form) and TCF4, whereas the level of total β-catenin remained unchanged (Figure 1E).

### ROS generation is critical to effect an acute Cr(VI)-induced miR-21 –PDCD4 signaling cascade

**Figure 2:**
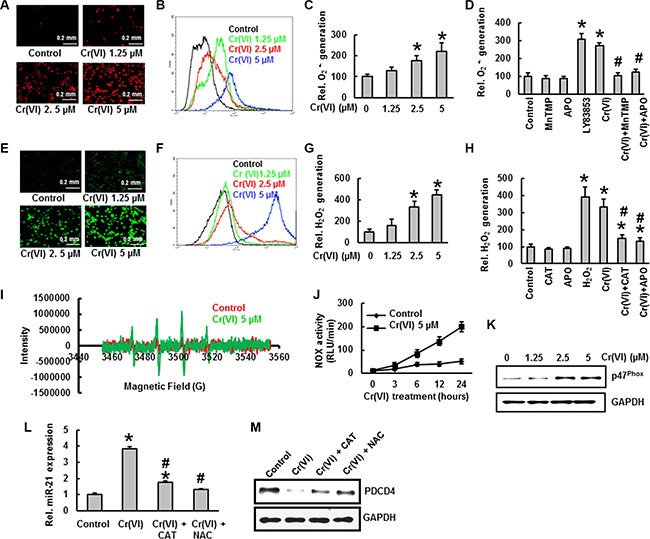
ROS generation is critical to effect an acute Cr(VI)-induced miR-21 –PDCD4 signaling cascade Cr(VI) induces ROS generation. Generation of O_2_ − and H_2_O_2_ were determined by staining the cells with DHE and DCFDA, respectively. BEAS-2B cells were exposed to Cr(VI) (0 to 5 μM) for 12 h and then were labeled with (**A**–**C**) DHE (10 μM) or (**E**–**G**) DCFDA (10 μM). Images were obtained by fluorescence microscopy and fluorescent intensity was determined by flow cytometry. BEAS-2B cells were exposed to Cr(VI) (0 or 5 μM) or were pretreated with MnTMPyP (5 μM; O_2_.- scavenger), CAT (1000 U/ml; H_2_O_2_ scavenger), or APO (50 μM; NOX inhibitor) for 2 h followed by Cr(VI) (5 μM) treatment for 6 h and then were labeled with (**D**) DHE (10 μM) or (**H**) DCFDA (10 μM) as described in the Materials and Methods section. LY83853 (10 μM) and H_2_O_2_ (0.1 mM) were used as positive controls for DHE and DCFDA measurements, respectively. (**I**) Generation of. OH was determined by ESR. (**J**) NOX activity was measured by the lucigenin chemiluminescence assay. (**K**) Cr(VI) increases the protein levels of NOX subunit, p47^phox^. Exogenous addition of ROS inhibitors catalase or NAC inhibited the acute Cr(VI)- induced (**L**) miR-21 increase and (**M**) PDCD4 suppression. Data presented in bar graphs are the mean ± SD of three independent experiments. *and ^#^indicate statistically significant differences compared to control without treatment or H_2_O_2_ /LY83853/ Cr(VI) treatment, respectively with *p* < 0.05.

A critical question for this investigation was whether Cr(VI)-induced ROS plays any role in miR-21 –PDCD4 signaling. Cr(VI)-induced ROS production was quantified by flow cytometry using the fluorescent probes DHE and DCFDA. Cr(VI) exposure dramatically stimulated O_2_ − and H_2_O_2_ generation in BEAS-2B cells, as indicated by an increase of DHE (Figure 2A–2C) and DCFDA (Figure 2E–2G) fluorescence intensity, respectively, when levels were compared to those generated from untreated control cells. The DHE signal was increased by Cr(VI) and LY83853 (O_2_ − donor) and inhibited by MnTMPyP, cell-permeable SOD mimetic (O_2_ − scavenger) (Figure 2D). Similarly, the DCFDA signal was increased by Cr(VI) and H_2_O_2_, and inhibited by CAT (H_2_O_2_ scavenger) (Figure 2H). The fluorescence intensity stimulated by Cr(VI) was also abolished by apocynin (APO), a NOX inhibitor. Further, the Cr(VI)-induced ^·^OH generation in BEAS-2B cells was detected by Electron spin resonance (ESR) (Figure 2I). As shown in Figure 2J, Cr(VI) exposure induced a drastic increase in NOX activity within 6 h and lasted for up to 24 h. Moreover we found that acute Cr(VI) treatment also increased the expression of p47phox, one of the NOX subunits (Figure 2K). Taken together, these results suggest that Cr(VI) exposure induces ROS production in BEAS-2B cells, and activation of NOX is required for this ROS generation.

Next we sought the role of Cr(VI)-induced ROS generation in miR-21- PDCD4 signaling. As outlined above, we demonstrated that ROS plays an essential role in Cr(VI)-induced miR-21 elevation and PDCD4 suppression. However, the Cr(VI)-induced miR-21 elevation and PDCD4 suppression were markedly reversed by treatment with the ROS inhibitors, NAC or Catalase (Figure 2L–2M). These results provide solid evidence that ROS plays a key role in Cr(VI)-induced miR-21 elevation and PDCD4 reduction.

### STAT3 binding to the miR-21 promoter upon IL-6 induction by Cr(VI)

**Figure 3:**
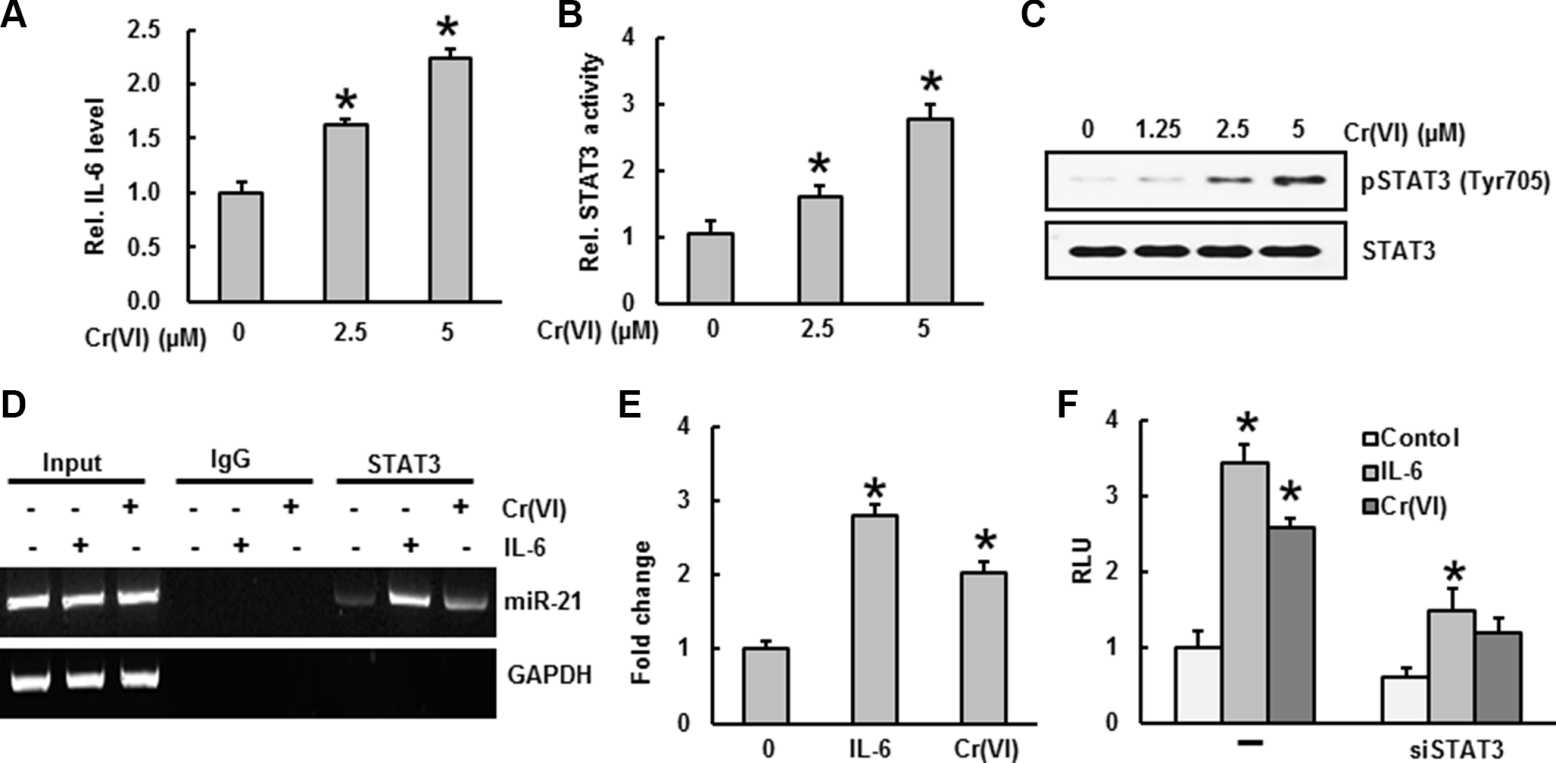
STAT3 binding to the miR-21 promoter upon IL-6 induction by Cr(VI) BEAS-2B cells were treated with Cr(VI) (2.5 and 5 μM) for 24 h. Culture medium was collected to estimate the (**A**) IL-6 level using commercially available ELISA kit according to manufacturer's recommendation. (**B**) STAT3 activity was measured in the nuclear fraction of cell lysates using ELISA following the manufacturer's protocol. (**C**) Total cell lysates were prepared for Western blot analysis using specific antibodies against pSTAT3 and STAT3. (**D**–**E**) BEAS-2B cells were exposed with IL-6 or Cr(VI) for 30 minutes and subjected to a chromatin immunoprecipitation (ChIP) analysis using anti-STAT3 or IgG isotype control. Coimmunoprecipitated DNA was amplified by PCR with primers specific for the miR-21 upstream enhancer. (**F**) Reporter gene assays were performed in BEAS-2B cells transfected either with a luciferase vector driven by the miR-21 promoter/enhancer alone (−) or in the presence of a vector encoding a small hairpin RNA silencing STAT3 expression (siSTAT3). Data presented in the bar graphs are the mean ± SD of three independent experiments. *indicates a statistically significant difference compared to control with *p* < 0.05.

Previous studies showed that Cr(VI) stimulates IL-6 mRNA levels and STAT3 phosphorylation in human airway epithelial cells [34]. STAT3 binding to the miR-21 promoter upon IL-6 induction has been reported previously [30]. To verify that IL-6 induces STAT3 binding to the miR-21 promoter after Cr(VI) treatment, we analyzed IL-6 levels and STAT3 activation by ELISA, and STAT3 binding to the miR-21 promoter by ChIP assay. Acute Cr(VI) treatment to BEAS-2B cells significantly (*p* < 0.05) increased the IL-6 levels (Figure 3A), STAT3 activation (Figure 3B), and STAT3 phosphorylation (Figure 3C) in a dose-dependent manner. In addition, Cr(VI) treatment also increased the binding of STAT3 on miR-21 promoter (Figure 3D–3E). A genomic DNA fragment extending from −1120 to +25 bp relative to the miR-21 transcription start site containing the putative STAT3 enhancer region proved to be IL-6–responsive in a reporter assay [30]. As shown in Figure 3F, miR-21 promoter activity was significantly (*p* < 0.05) elevated by Cr(VI) or IL-6, while STAT3 knock down (siSTAT3) inhibited miR-21 promoter activity.

### miR-21 elevation and PDCD4 suppression contribute to Cr(VI)-induced malignant cell transformation

**Figure 4:**
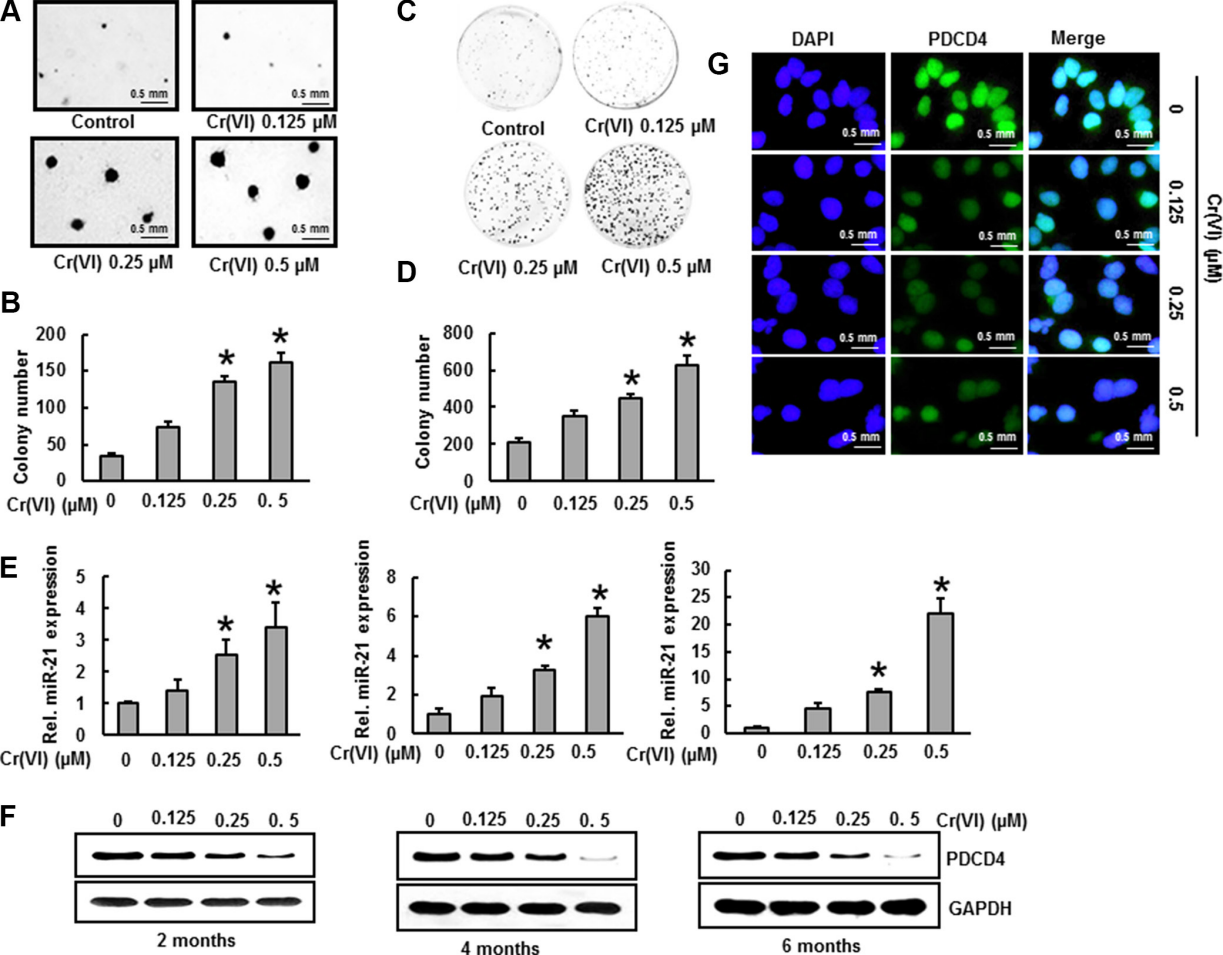
Cr(VI)-induced miR-21 increase and PDCD4 suppression contribute to malignant cell transformation BEAS-2B cells were maintained in a medium containing various concentrations of Cr(VI) (0.125, 0.25 and 0.5 μM) for 6 months. (**A**–**B**) Cells were cultured in 0.35% soft agar for 5 weeks. The number of colonies in the entire dish was counted. (**C**–**D**) Cells (300 cells) from indicated Cr(VI) treatments were seeded into each of three dishes (60 mm diameter), and grown for an additional 10 days and stained with crystal violet. Colony numbers in the entire dish were counted. (**E**) The relative miR-21 level was determined by Taqman real-time PCR. (**F**) PDCD4 protein levels after chronic Cr(VI) treatment was detected by immunoblot analysis. The three panels show data obtained at 2, 4 and 6 months of treatment. (**G**) Representative images of fluorescence immunostaining for PDCD4. Data presented in the bar graphs are the mean ± SD of three independent experiments. *indicates a statistically significant difference compared to control with *p* < 0.05.

Chronic Cr(VI) exposure induces malignant transformation in BEAS-2B cells [3]. Malignant cell transformation was assessed by anchorage-independent growth in soft agar [35]. Long term (6 months) treatment with low concentrations (0.125, 0.25 and 0.5 μM) of Cr(VI) induced malignant transformation of BEAS-2B cells as shown by the marked increase in size and number of colonies compared to untreated control (Figure 4A–4B). A similar trend was observed with the clonogenic assay, where chronic Cr(VI) exposure to BEAS-2B cells showed a significant (*p* < 0.05) and dose-dependent increase in colony number (Figure 4C–4D).

We treated BEAS-2B cells with various concentrations (0.125, 0.25 and 0.5 μM) of Cr(VI) and measured the miR-21 levels and PDCD4 expression at two, four and six months. We found a dose-dependent and significant (*p* < 0.05) increase in the miR-21 levels (Figure 4E) associated with a dose-dependent and drastic decrease in the PDCD4 expression (Figure 4F) with chronic Cr(VI) exposure. Similar results were observed by immunofluorescence, when BEAS-2B cells were treated with Cr(VI) for six months and showed a marked decrease in the PDCD4 level (Figure 4G). These observations clearly emphasize a role for miR-21-PDCD4 signaling in Cr(VI)-induced transformation.

### PDCD4 suppression by Cr(VI) down-regulates E-cadherin and increases the accumulation of β- catenin and TCF4 in the nucleus

**Figure 5:**
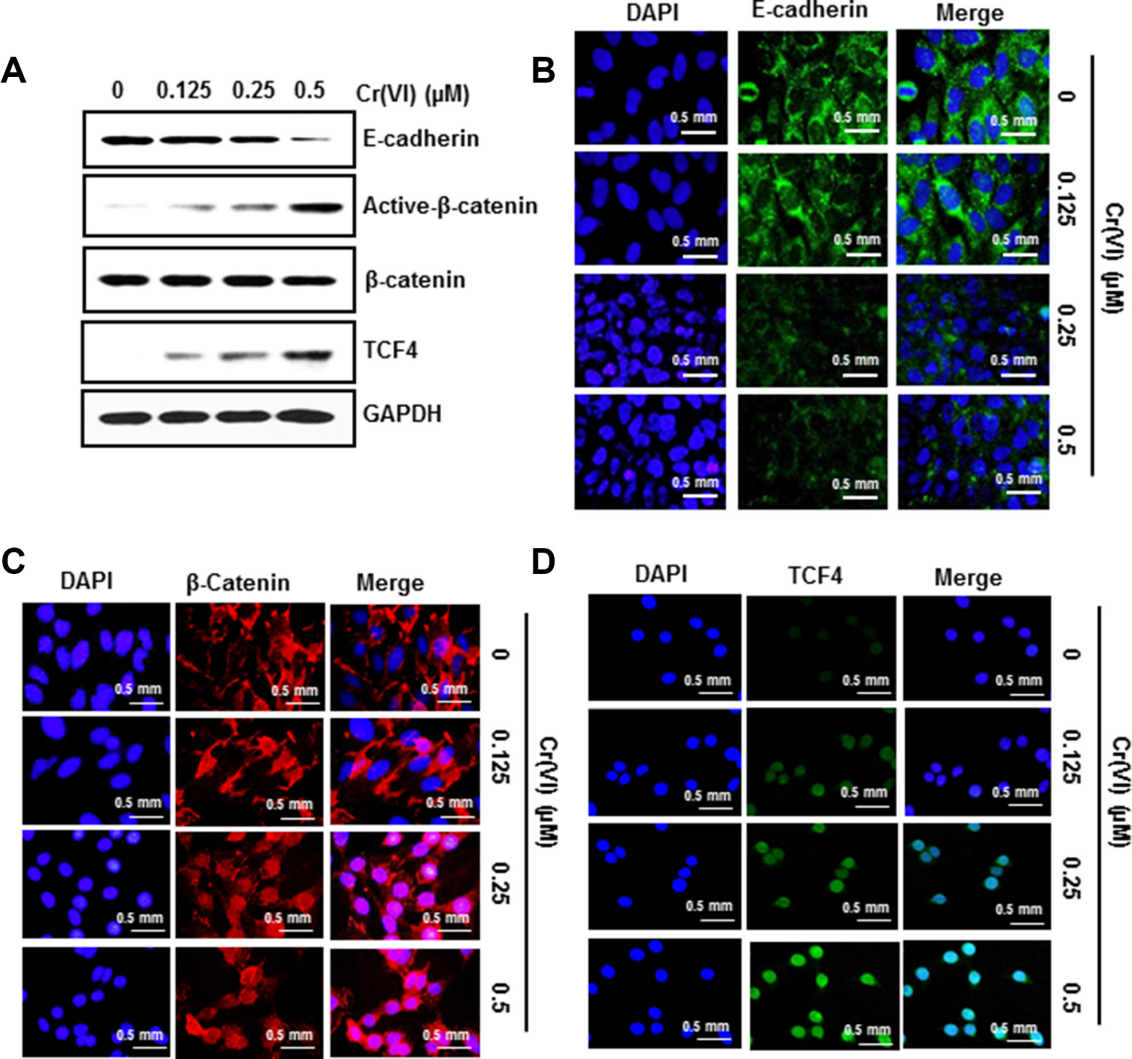
PDCD4 suppression by Cr(VI) down-regulates E-cadherin and increases the accumulation of β- catenin and TCF4 in the nucleus BEAS-2B cells were maintained in a medium containing various concentrations of Cr(VI) (0.125, 0.25 and 0.5 μM) for 6 months. (**A**) Total cell lysates were prepared for Western blot analysis using specific antibodies against E-Cadherin, Active-β-catenin, β-catenin, TCF4 and GAPDH. Representative images of fluorescence immunostaining of (**B**) E-cadherin (**C**) β- catenin and (**D**) TCF4.

Previous studies reported that suppression of PDCD4 elicits an inhibition of E-cadherin expression, which is associated with an increase in β- catenin activation and TCF4 expression [26]. To evaluate this, we treated BEAS-2B cells with different concentrations of Cr(VI) for six months. As shown in Figure 5A, chronic Cr(VI) exposure resulted in decreased E-cadherin protein levels, while active β- catenin and TCF4 expressions increased, and these effects occurred in a dose-dependent manner. In confirmation, immunofluorescence analysis indicated that chronic Cr(VI) exposure decreased E-cadherin expression (Figure 5B) and increased β- catenin and TCF4 accumulation in the nucleus (Figure 5C–5D). These data clearly show that chronic Cr(VI) exposure suppresses E-cadherin and increases the accumulation of β- catenin and TCF4 in the nucleus during malignant transformation.

### PDCD4 suppression by Cr(VI) increases the expression of uPAR and c-Myc

**Figure 6:**
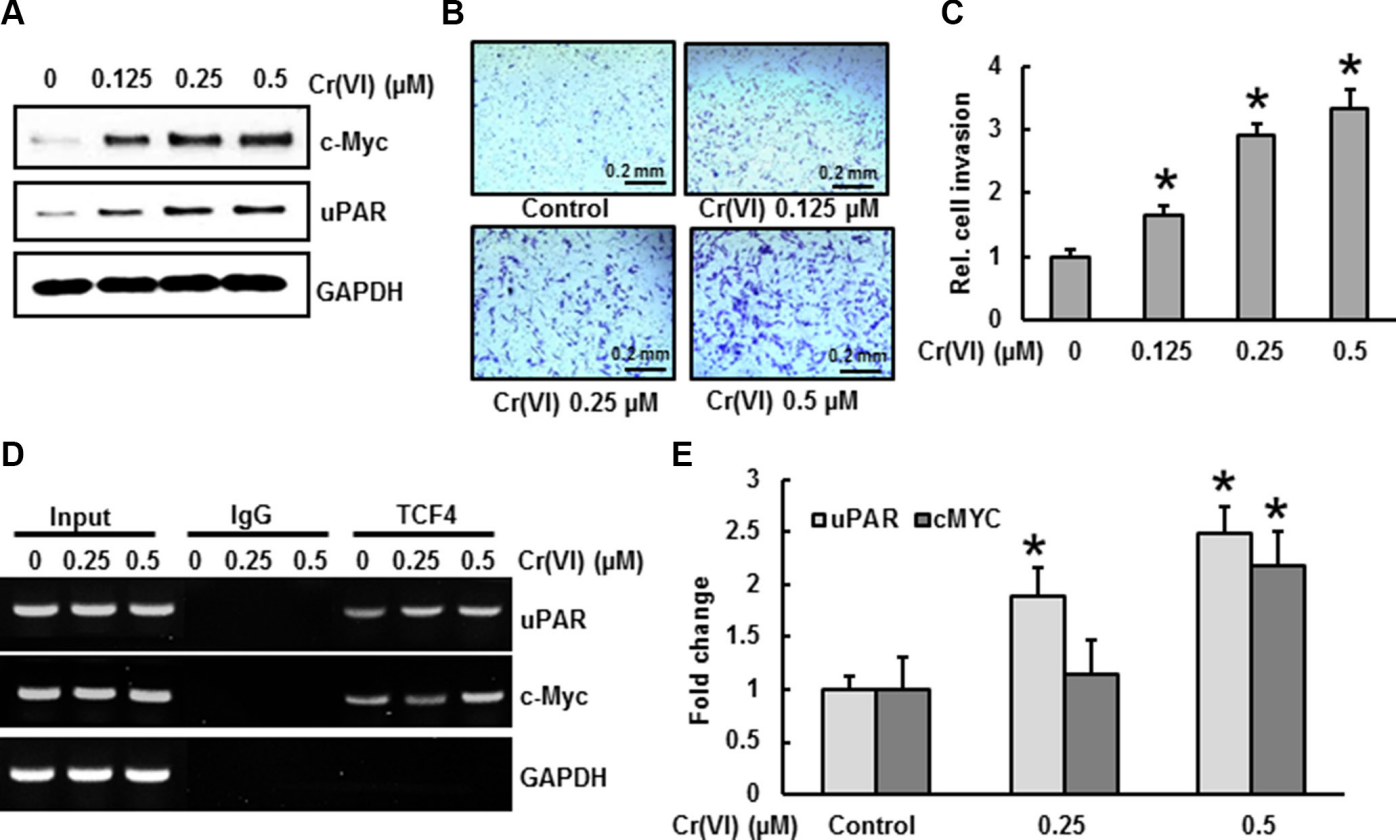
PDCD4 suppression by Cr(VI) increases the expression of uPAR and c-Myc BEAS-2B cells were exposed to various concentrations of Cr(VI) (0.125, 0.25 and 0.5 μM) for 6 months. (**A**) Expression levels for uPAR and c-Myc were analyzed by Western blot. To evaluate invasive capacity (**B**–**C**) 1 × 10^5^ cells from each group were seeded on the top chamber of 24-well plate culture inserts coated with 20 μl of matrigel in duplicate. Cells were cultured for additional 48 h. Invaded cells on the bottom of insert were stained and quantified. (**D**–**E**) For the ChIP assay, TCF4 binding regions on uPAR and c-Myc promoters were identified. Chromatin isolated from chronic Cr(VI) exposed BEAS-2B cells were immunoprecipitated with an anti-TCF4 antibody or control mouse IgG. The TCF4 binding to the uPAR and c-Myc promoters was analyzed by RT PCR with specific primers. Data presented in the bar graphs are the mean ± SD of three independent experiments. *indicates a statistically significant difference compared to control with *p* < 0.05.

The c-Myc oncogene plays a vital role at both early and late stages of carcinogenesis [36]. uPAR is involved in cancer cell invasion and significantly correlates to tumor aggressiveness and poor outcome [37]. It has been reported that uPAR and c-Myc are the target genes of β-catenin/TCF4 dependent transcription [26]. As shown in Figure 6A, chronic Cr(VI) exposure increased c-Myc and uPAR expressions in BEAS-2B cells in a dose-dependent manner. Similarly, the invasive potential of BEAS-2B cells under chronic Cr(VI)-exposure was also increased in correlation with uPAR expression (Figure 6B–6C). BEAS-2B cells treated with 0.5 μM Cr(VI) for 6 months showed a 3-fold increase for invasion compared to untreated control cells (Figure 6C). A previous study demonstrated that in PDCD4 knock down cells, β-catenin binds with TCF4 in the nucleus, with subsequent binding of β-catenin/TCF4 complex to the uPAR and c-Myc promoters [26]. To investigate this mechanism for Cr(VI)-induced carcinogenesis, we performed ChIP analysis using a TCF4 antibody and primers that specifically amplify the β-catenin/TCF4 binding site on the promoters of uPAR (−308 to −302) and c-Myc (−452 to −446). As shown in Figure 6D–6E, association of the β-catenin/TCF4 complex to the uPAR and c-Myc promoters increased during chronic Cr(VI) treatment in a dose-dependent manner. These results indicate that PDCD4 suppression increases uPAR and c-Myc expressions, and also activates β-catenin/TCF4 complex binding to the uPAR and c-Myc promoters during chronic Cr(VI) exposure.

### Stable knockdown of miR-21 and overexpression of PDCD4 inhibit Cr(VI)-induced malignant cell transformation and invasion

**Figure 7:**
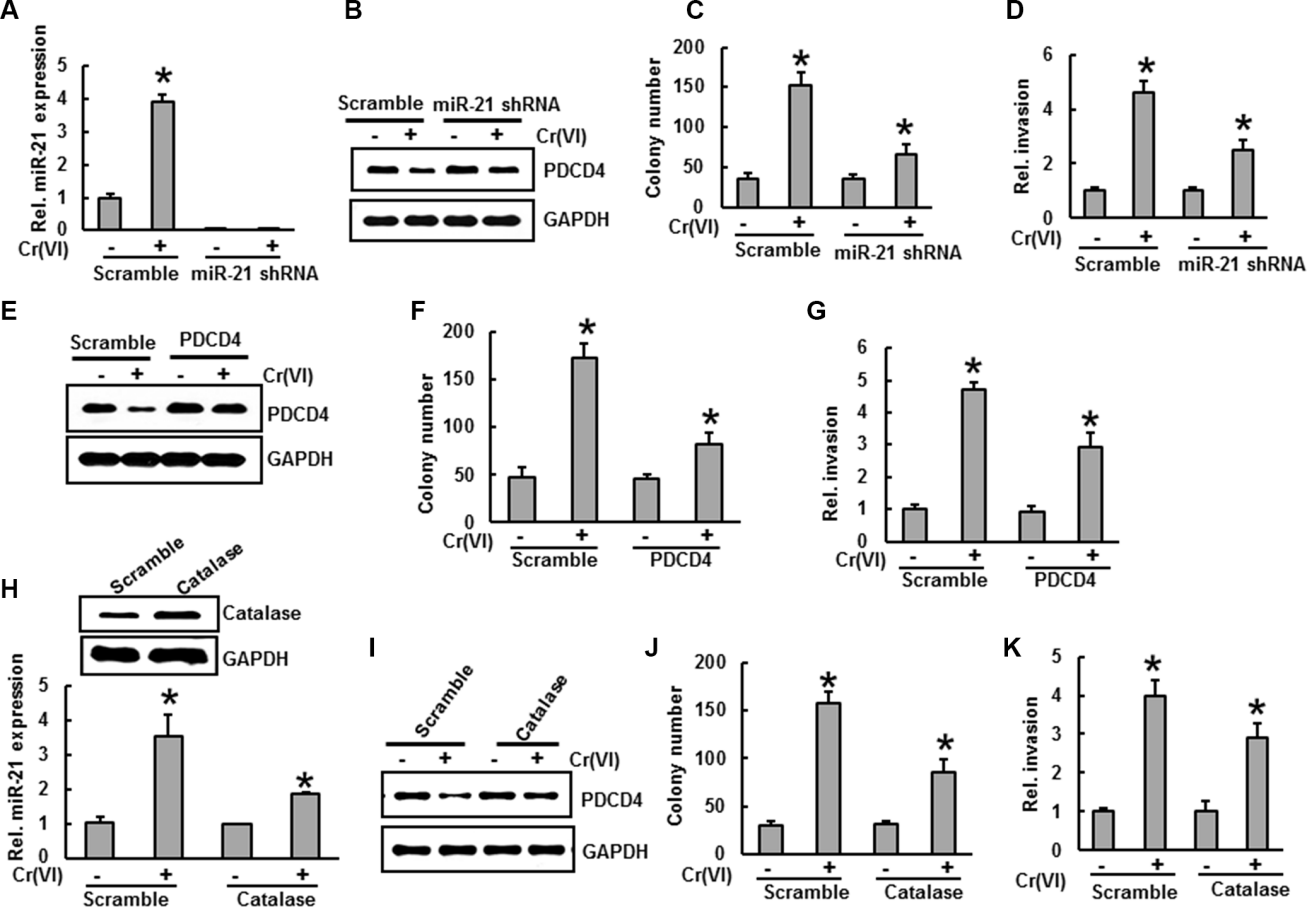
Stable knockdown of miR-21 and overexpression of PDCD4 or catalase inhibit Cr(VI)-induced malignant cell transformation and invasion (**A**–**D**) BEAS-2B cells were stably knockeddown with miR-21 shRNA or their corresponding vehicle vector and exposed to Cr(VI) (0 or 0.5 μM) for 6 months. (A) the relative miR-21 level was determined by Taqman real-time PCR. (B) Cell lysates were prepared to determine the protein level of PDCD4 using Western blot analysis. (C) Anchorage-independent colony growth was assessed as previously described. (D) Cell invasion was assessed as described previously (E-G) BEAS-2B cells were stably overexpressed with PDCD4 or their corresponding vehicle vector and exposed with Cr(VI) (0 or 0.5 μM) for 6 months. (**E**) Cell lysates were prepared to determine the protein level of PDCD4 by Western blot analysis. (**F**) Anchorage-independent colony growth and (**G**) cell invasion were assessed as previously described. (H-K) BEAS-2B cells were stably overexpressed with catalase or their corresponding vehicle vector and exposed with Cr(VI) (0 or 0.5 μM) for 6 months. (**H**) The relative miR-21 level was determined by Taqman real-time PCR. (**I**) PDCD4 protein levels after chronic Cr(VI) treatment was detected by immunoblot analysis. (**J**) Anchorage-independent colony growth and (**K**) cell invasion were assessed as previously described. Data presented in the bar graphs are the mean ± SD of three independent experiments. * indicates a statistically significant difference compared to control with *p* < 0.05.

To determine the oncogenic role of miR-21 in Cr(VI)-induced malignant transformation and invasion, BEAS-2B cells with stable knocked down of miR-21 were treated with Cr(VI) (0.5 μM) for six months. As shown in Figure 7A, miR-21 knock down cells showed no increase in miR-21 levels even after chronic Cr(VI) treatment. BEAS-2B cells with miR-21 knock down also blocked the suppression of its target tumor suppressor, PDCD4 that normally occurs with chronic Cr(VI) treatment (Figure 7B). Importantly, miR-21 knock down significantly inhibited the chronic Cr(VI)-induced malignant cell transformation (Figure 7C) and invasion (Figure 7D). Similarly over-expression of PDCD4 (Figure 7E) in BEAS-2B cells significantly (*p* < 0.05) decreased the chronic Cr(VI)-induced malignant cell transformation (Figure 7F) and invasion (Figure 7G). These results demonstrated that the increased levels of oncomiR miR-21 and suppression of tumor suppressor PDCD4 are critically important for Cr(VI)-induced malignant cell transformation and invasion.

### Antioxidants inhibit chronic Cr(VI)-induced miR-21 elevation and PDCD4 suppression

To investigate the role of antioxidants on chronic Cr(VI)-induced miR-21 elevation and PDCD4 suppression, we treated BEAS-2B cells overexpressing catalase with Cr(VI) (0.5 μM) for six months. Our study shows that overexpression of catalase noticeably decreased the miR-21 elevation induced by chronic Cr(VI) treatment (Figure 7H) and suppressed the PDCD4 reduction (Figure 7I) in BEAS-2B cells with chronic Cr(VI) treatment. Furthermore, catalase overexpression also decreased the chronic Cr(VI)-induced malignant cell transformation (Figure 7J) and invasion (Figure 7K). These observations clearly demonstrate the indispensable role of ROS in regulating miR-21-PDCD4 signaling during chronic Cr(VI)-induced malignant cell transformation and invasion.

### Elevated miR-21 and suppressed PDCD4 expression in Cr(VI)-exposed animals and xenograft tumors

**Figure 8:**
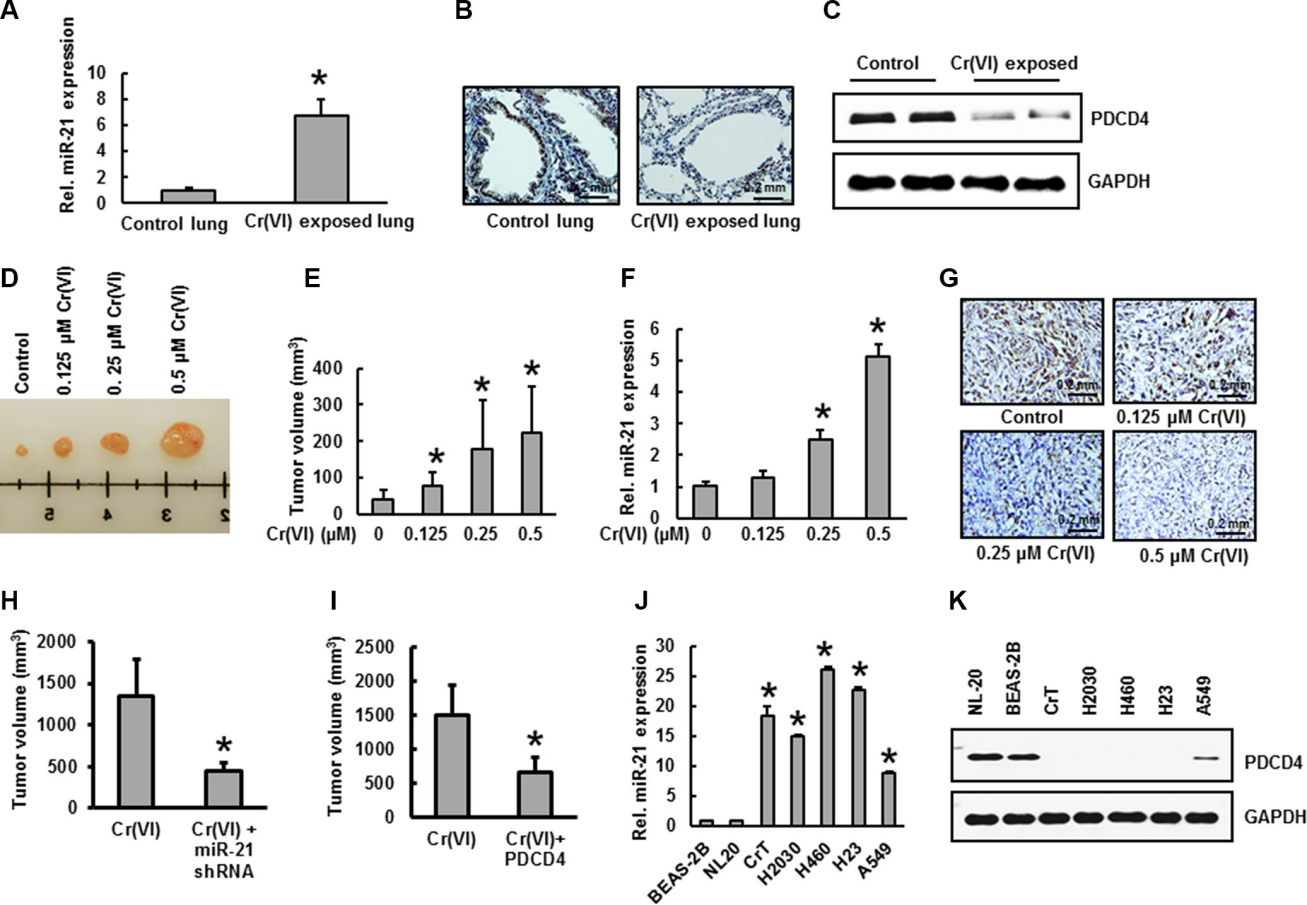
Increased miR-21 and suppressed PDCD4 expression in Cr(VI)-exposed animals and xenograft tumors (**A**–**C**) Female BALB/c mice were treated intranasally with either insoluble Cr (VI) (1.2 mg/ml) or PBS (vehicle control), once a day, 5 days/week. After 3 months, mice were euthanized using CO_2_ and lung tissue was isolated for further examination. (A) The relative miR-21 level was determined by Taqman real-time PCR. PDCD4 protein expression was detected by (B) immunohistochemistry and (C) immunoblot analysis. (**D**–**G**) Nude mice were injected sc with BEAS-2B cells exposed to indicated concentration of Cr(VI) for 6 months. After 4 weeks, mice were euthanized using CO_2_ and tumor was isolated for further examination. (D–E) Tumor volume was measured and (F) the relative miR-21 level was determined by Taqman real-time PCR. (G) PDCD4 protein expression was detected by immunohistochemistry. (H-I) Inhibition of *in vivo* tumor growth in nude mice with miR-21 knockdown and PDCD4 overexpression in chronic Cr(VI)-exposed BEAS-2B cells. BEAS-2B cells with (**H**) miR-21 knockdown, (**I**) PDCD4 overexpression and respective vector controls were exposed to Cr(VI) (0 or 0.5 μM) for 6 months, xenograft growth of tumors in nude mice was performed as described previously. (**J**–**K**) Different lung cancer cell lines were used to determine the basal levels of (J) miR-21 and (K) PDCD4 expression. Data presented in the bar graphs are the mean ± SD of three independent experiments. * indicates a statistically significant difference compared to control with *p* < 0.05.

To explore whether Cr(VI) exposure causes miR-21 elevation and PDCD4 suppression *in vivo*, BALB/c mice were nasally exposed to insoluble Cr(VI) particles (1.2 mg/ml) for up to 3 months. The results show that chronic Cr(VI) exposure at 1.2 mg/ml induced an increase in miR-21 levels (Figure 8A) and decreased PDCD4 expression (Figure 8B–8C) in mouse lung tissue compared to control. Next we investigated tumorogenicity in nude mice using BEAS-2B cells chronically exposed to Cr(VI). For this study, nude mice were injected sc with BEAS-2B cells that had been exposed to Cr(VI) for 6 months at the indicated concentration. Over a 4-week period post inoculation, we observed visible tumor formation that increased progressively in size for mice injected with Cr(VI)-treated BEAS-2B cells but not in those injected with control cells (Figure 8D–8E). Consistent with our *in vitro* findings above, we found significantly increased miR-21 levels (Figure 8F) associated with decreased PDCD4 expression (Figure 8G) in xenograft tumors generated with chronic Cr(VI) exposed BEAS-2B cells. In addition we also demonstrated that stable knockdown of miR-21 or overexpression of PDCD4 in BEAS-2B cells chronically exposed to Cr(VI) reduced tumorogenicity in nude mice. For these studies, BEAS-2B cells stably expressing miR-21 shRNA, PDCD4, or vector, with or without Cr(VI) exposure for 6-month, were injected to nude mice. Four weeks after injection, tumor development was assessed. Tumors developed from BEAS-2B cells with stable knockdown of miR-21 and exposed to Cr(VI) (Figure 8H), and tumors formed from Cr(VI)-exposed BEAS-2B cells overexpressing PDCD4 (Figure 8I) were smaller than those of Cr(VI)-exposed-vector cells. To confirm these results, we examined the basal levels of miR-21 and PDCD4 in a variety of lung cancer cell lines. Our results showed increased miR-21 levels (Figure 8J) and decreased PDCD4 expression (Figure 8K) in the lung cancer cells (H2030, H460, H23, and A549) compared to normal lung epithelial cells (BEAS-2B and NL-20).

### The levels of miR-21 increase and PDCD4 decrease in human lung adenocarcinoma tissue

**Figure 9:**
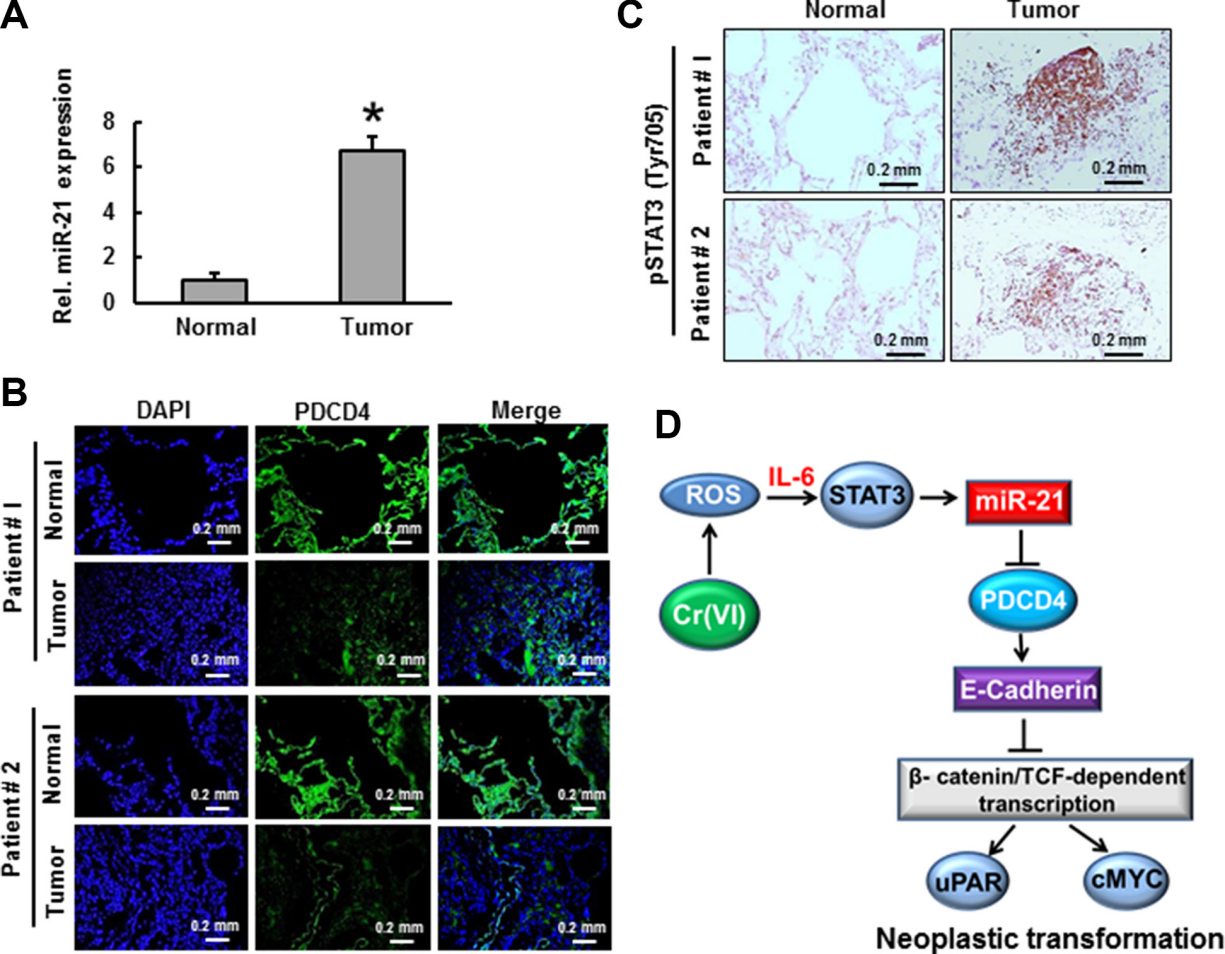
The levels of miR-21 and PDCD4 in lung adenocarcinoma Lung adenocarcinoma tissues from patients was used to demonstrate the (**A**) relative miR-21 level compared to normal tissue from same patients. (**B**) PDCD4 protein expression was detected by immunofluorescence and (**C**) STAT3 phosphorylation was determined by immunohistochemistry. Data presented in the bar graphs are the mean ± SD of three independent experiments. *indicates a statistically significant difference compared to control with *p* < 0.05. (**D**) Proposed mechanism of Cr(VI)-induced malignant cell transformation and carcinogenesis.

The expression levels of miR-21 and its downstream target protein PDCD4 were determined by real time PCR and florescence immunohistochemistry respectively in lung tissues from lung adenocarcinoma patients. As shown in Figure 9A, there was a 6-fold increase in miR-21 level in lung cancer tissues compared to the normal tissues from same patients. However, the expression levels of PDCD4 were decreased in all lung cancer tissues when compared with normal tissues as determined by florescence immunohistochemistry (Figure 9B). Moreover, phosphorylated STAT3 was also increased in lung tumor tissues of patients (Figure 9C). These results demonstrate the indispensable role of the miR-21-PDCD4 signaling axis in lung carcinogenesis.

## DISCUSSION

Occupational exposure to Cr(VI) is a well-established cause of lung cancer [38–41], therefore, Cr(VI) is considered a class I carcinogen [42]. Several studies have shown that ROS generated by Cr(VI) exposure is one of the major causes of carcinogenesis [3, 45, 43, 44]. Although Cr(VI) is a well-established carcinogen, relatively little is known about the molecular mechanisms of Cr(VI)-induced malignant transformation. In this study we detail a mechanism for Cr(VI)-induced malignant transformation and the involvement of a miR-21-PDCD4 signaling process.

MicroRNA's (miRs) are important post-transcriptional regulators that influence more than 30% of human mRNAs. Significant changes in microRNA (miR) expression in response to environmental exposure in humans have recently been reported [46, 47]. Notably miR-21, a key oncogene, plays an important role in the initiation and progression of cancer [47] and is highly overexpressed in most cancers [48, 49]. miR-21 binds to the 3′-UTR of the tumor suppressor PDCD4 to suppress its translation [4]. Down-regulation of PDCD4 expression by miR-21 leads to tumor cell growth, survival, chemoresistance, invasion and metastasis [4, 50]. Inhibition of miR-21 or overexpression of PDCD4 results in decreased tumor formation and growth [51]. Therefore, miR-21 and PDCD4 are potential targets for novel cancer prevention and therapeutics. In our preliminary analysis, we found increased miR-21 levels and decreased PDCD4 expressions in lung cancer cell lines (H2030, H460, H23, and A549) compared to those found in normal lung epithelial cells (BEAS-2B and NL-20). We investigated the effect of Cr(VI) on the interaction of miR-21-PDCD4 signaling and subsequent malignant cell transformation; both acute and chronic treatments of Cr(VI) induce miR-21 increases with an associated suppression of PDCD4. Cr(VI) also increased the binding of miR-21 to the 3′-UTR of PDCD4. Moreover, the increased miR-21 and reduced PDCD4 levels brought about by chronic Cr(VI) treatments in BEAS-2B cells also induced malignant transformation. Furthermore, stable shut down of miR-21 and overexpression of PDCD4 in BEAS-2B cells significantly inhibited the chronic Cr(VI)-induced malignant transformation. These results strongly demonstrate that this interactive miR-21-PDCD4 signaling plays an important role in Cr(VI)-induced malignant transformation.

We also explored the mechanisms by which Cr(VI) activates miR-21. STAT3, known to be important for cancer initiation and tumor progression, was reported to bind directly to the miR-21 promoter upon IL-6 induction [28, 30, 52]. In our study treatment of BEAS-2B cells with Cr(VI) significantly increased IL-6 secretion. In addition Cr(VI) also triggered STAT3 transcriptional activation and phosphorylation. Furthermore, the binding of STAT3 on the miR-21 promoter was also increased with Cr(VI) treatment. These data clearly suggest that Cr(VI)-induced IL-6 secretion mediates STAT3 activation, which is crucial for the rise in miR-21.

Studies suggest that hydrogen peroxide (H_2_O_2_) can up-regulate miR-21 levels and decrease PDCD4 expression in vascular smooth muscle cells [53]. It is also established that Cr(VI)-induced ROS is crucial to malignant cell transformation [3]. However, the role of Cr(VI)-induced ROS in miR-21-PDCD4 signaling and malignant transformation is currently unclear. We demonstrated that Cr(VI) induces ROS and that p47^phox^, one of the NOX subunits is the key source Cr(VI)-induced ROS. Moreover, exogenous treatment of cells with catalase or NAC significantly inhibited the Cr(VI)-induced a rise in miR-21 and decreased PDCD4 levels. To substantiate a role for antioxidants in the Cr(VI)-induced malignant cell transformation mediated by increased miR-21 levels and PDCD4 suppression, we chronically treated BEAS-2B cells overexpressing catalase with Cr(VI) for six months. Interestingly, catalase overexpression in BEAS-2B cells significantly inhibited Cr(VI)-induced miR-21 elevation, PDCD4 suppression and malignant cell transformation.

A disturbance in epithelial cell adhesion, which leads to a more invasive and metastatic phenotype, is a hallmark of tumor progression [54]. E-cadherin, a well-known primary transmembrane glycoprotein molecule is involved in Ca^2+^ -dependent cell–cell adhesion in epithelial tissues. Several studies report a strong correlation between E-cadherin loss and the initiation and progression of tumors [26, 54]. The cytoplasmic domain of E-cadherin is attached to actin microfilaments via two other molecules, α- and β-catenin [55]. Disruption of these E-cadherin homotypic interactions results in the release of β -catenin from E-cadherin and subsequent translocation of β -catenin into the nucleus; the nuclear β-catenin associates with TCF4 (a member of transcription factor family) to form a β-catenin/TCF complex and activates the transcription of genes involved in cancer initiation, progression and metastasis such as c-Myc, and uPAR [26]. Down-regulation of PDCD4 leads to an inhibition of E-cadherin expression, which is associated with an increase in β- catenin/TCF4 dependent transcription [26]. In our study both acute and chronic treatment with Cr(VI) suppressed the E-cadherin expression, and was accompanied by an increased active β- catenin and TCF4 expression in BEAS-2B cells in a dose-dependent manner. In addition, chronic Cr(VI) exposure increased uPAR and c-Myc protein expressions in BEAS-2B cells, as well as their invasive potential. Furthermore, chronic Cr(VI) exposure increased the binding of TCF4 on uPAR and c-Myc promoters in BEAS-2B cells. These findings indicate that PDCD4 suppression is critical for the β-catenin/TCF dependent transcription of c-Myc, and uPAR in Cr(VI)-induced carcinogenesis.

To explore whether Cr(VI) exposure causes miR-21 elevation and PDCD4 suppression *in vivo*, BALB/c mice were nasally exposed to insoluble Cr(VI) particles for 3 months. We found an increase in miR-21 levels associated with PDCD4 suppression in the lungs of mice exposed to Cr(VI). Moreover, we found an significantly increased miR-21 levels associated with decreased PDCD4 expression in xenograft tumors generated with chronic Cr(VI) exposed BEAS-2B cells. In addition we confirmed that stable knockdown of miR-21 or overexpression of PDCD4 reduce the tumorigenicity of chronic Cr(VI) exposed BEAS-2B cells in nude mice. Similar results were observed in lung adenocarcinoma tissues from patients compared with normal tissues from same patients.

In summary, we found that chronic Cr(VI) exposure increases miR-21 levels with an associated inhibition of PDCD4 expression, and cause malignant transformation in BEAS-2B cells. Cr(VI) triggers the miR-21 increase via the IL-6/STAT3 pathway. Cr(VI)-induced PDCD4 suppression down-regulates the E-cadherin protein expression along with an upregulation of active β-catenin (nuclear translocated form) and TCF4 in BEAS-2B cells. In addition, Cr(VI) exposure increases uPAR and c-Myc expressions, and also activates β-catenin/TCF4 complex binding to the uPAR and c-Myc promoters. Stable knockdown of miR-21 or overexpression of PDCD4 reduces the tumorogenicity of chronic Cr(VI) exposed BEAS-2B cells in nude mice (Figure 9D). In short, our findings demonstrate an indispensable role for an miR-21-PDCD4 signaling axis in Cr(VI)-induced malignant cell transformation and lung carcinogenesis.

## MATERIALS AND METHODS

### Antibodies and chemicals

Potassium dichromate (K_2_Cr_2_O_7_), apocynin (APO), 5,5-dimethyl-1-pyrroline-1-oxide (DMPO), were purchased from Sigma-Aldrich (St Louis, MO). Both Dichlorodihydrofluoresceine acetate (DCFDA) and dihydroethidium (DHE) were purchased from Molecular Probes (Eugene, OR). Manganese(III) tetrakis(1-methyl-4-pyridyl) porphyrin (MnTMPyP) was purchased from Cayman Chemical (Ann Arbor, MI). Human miR-21 hairpin inhibitor was purchased from Thermo Fisher Scientific Dharmacon (Chicago, IL, USA). Antibodies against PDCD4 (CST#9535), E-cadherin (CST#3195), β-catenin (CST#8480), c-Myc (CST#5605), uPAR (CST#9692), pSTAT3 Tyr705 (CST#9145) and STAT3 (CST#9139) were purchased from Cell Signaling Technology (Danvers, MA). Anti–active-β-catenin (clone 8E7#05-665) antibody was purchased from EMD Millipore (Billerica, MA). Antibodies against TCF4 (sc-13027) and GAPDH (sc-25778) were purchased from Santa Cruz Biotechnology, Inc. (Santa Cruz, CA).

### Cell lines and cell culture

Human bronchial epithelial cell lines BEAS-2B, NL20 and other lung cancer cell lines H2030, H460, H23, A549 cells were obtained from the American Type Culture Collection (Rockville, MD). Chromium transformed cells (CrT) were generated as described previously [45]. NL20 cells were grown at 37°C in humidified air containing 5% CO_2_ in serum-free LHC-9 medium. BEAS-2B and CrT cells were cultured in Dulbecco's modified Eagle's medium (DMEM), while H2030, H460, H23, and A549 cells were cultured in RPMI 1640 supplemented with 10% fetal bovine serum (FBS), 2 mM L-glutamine, and 5% penicillin/streptomycin at 37^°^C in a humidified atmosphere with 5% CO_2_ in air.

K_2_Cr_2_O_7_ was used for Cr(VI) treatments. For short-term exposure of Cr(VI), cells were grown to 80–90% confluent, and then the medium was replaced with DMEM medium containing 0.1% FBS for overnight before Cr(VI) treatment at the dose and duration indicated. In some experiments, cells were first pretreated with various inhibitors for 2 h and then exposed to Cr(VI). For chronic exposure of Cr(VI), the cells were continuously cultured in growth medium with the indicated concentration Cr(VI).

### Plasmids and transfection

Plasmid DNA encoding human catalase and STAT3 shRNA were purchased from Origene (Rockville, MD). PDCD4 3′-UTR reporter plasmid was kindly provided by Dr. Yong Li (University of Louisville, USA). The STAT3-miR-21 reporter was constructed as described previously [30]. PcDNA3.1/PDCD4 plasmid was kindly provided by Dr. Hsin-Sheng Yang (University of Kentucky, USA).

The overexpression of catalase or PDCD4 and knockdown of STAT3 in BEAS-2B cells were performed using Lipofectamine™2000 (Invitrogen, Carlsbad, CA) according to the manufacturer's protocol. Briefly, BEAS-2B cells were seeded at approximately 50% confluency in 6-well culture plates and transfected with 4 μg plasmid. Cell clones resistant to G418 were isolated; overexpression of CAT and PDCD4 protein production were confirmed by immunoblotting.

The pLenti-III-miR-Off-has-miR-21-puro-GFP expression vector and the negative control vector pLenti-III-miR-Off- puro-GFP were purchased from Applied Biological Materials, Inc. (Richmond, CA). Human embryonic kidney 293T cells (ATCC, Manassas, VA) were transfected with lentiviral packaging vectors (ABM, Richmond, BC, CA) and lentiviral vectors expressing miR-Off-has-miR-21-puro-GFP or miR-Off- puro-GFP by Lipofectamine™2000 (Invitrogen, Carlsbad, CA) according to the manufacturer's protocol [56].

For generating stable miR-21 knockdown cell lines, two days after transfection, supernatants containing viral particles were harvested and used to infect BEAS-2B cells at approximately 70% confluence in DMEM supplemented with 8 μg/ml of polybrene using lentifectin reagent (ABM technologies) following the manufacture's protocol. 72 h after transfection, the medium was changed to the selection DMEM with 10% FBS and supplemented with puromycin 1 μg/mL (sigma) to screen stable cell lines for further assay. Three weeks later, the cell clones were screened and further cultured in DMEM medium containing 1 μg/mL puromycin and selected through FACS (Fluorescent Assisted Cell Sorting system).

### Intracellular ROS determination

H_2_O_2_ and O_2_.- generations were examined using the fluorescent dye DCFDA and DHE, respectively, as described previously [45]. The cells were cultured in 6-well plates (2 × 10^5^ cells/well), treated with 5 μM of Cr(VI) for 12 h and incubated with DCFDA or DHE (10 μM) for 40 min at 37°C. Trypsinized cells were washed twice with cold PBS, and analyzed by fluorescence-activated cell sorting (FACS Calibur, BD Biosciences). The fluorescence intensity of DCFDA was measured at an excitation wavelength of 492 nm and an emission wave length of 517 nm. The fluorescence intensity of DHE was measured at an excitation wavelength of 535 nm and an emission wavelength of 610 nm.

### Electron spin resonance (ESR) assay

ESR measurement was conducted using a Bruker EMX spectrometer (Bruker Instruments, Billerica, MA) and a flat cell assembly. The intensity of ESR signal was used to measure the amount of hydroxyl radical generation. DMPO was used as a spin or radical trap. DMPO was charcoal purified and distilled to remove all ESR detectable impurities before use. Hyperfine couplings were measured (to 0.1 G) directly from magnetic field separation using potassium tetraperoxochromate (K_3_CrO_8_) and 1,1-diphenyl-2-picrylhydrazyl (DPPH) as reference standards. Reactants were mixed in test tubes to a total final volume of 0.5 mL. The reaction mixture was then transferred to a flat cell for ESR measurement.

### Clonogenic assay

BEAS-2B cells (10^5^ cells) were seeded into each well of a 6-well plate and allowed to attach overnight. After the indicated exposure, cells were collected by trypsinization, three hundred cells were then reseeded into each of three dishes (60 mm diameter), and grown for 10 days. The cells were fixed with 2% formalin for 10 min and stained with 0.5% crystal violet stain and counted.

### NOX activity assay

NOX activity was measured by the lucigenin enhanced chemiluminescence method as described previously [3]. Briefly, cells were harvested and homogenized by sonication in cold lysis buffer (20 mM KH_2_PO_4_, pH 7.0, 1 mM EGTA, 1 mM phenyl methyl sulfonyl fluoride, 10 μg/ml aprotinin, and 0.5 μg/ml leupeptin). Homogenates were centrifuged at 800 × g at 4^°^C for 10 min to remove the unbroken cells and debris, and aliquots were used immediately. To start the assay, 100-μl of homogenate supernatants were added to 900 μl of 50 mM phosphate buffer, pH 7.0, containing 1 mM EGTA, 150 mM sucrose, 5 μM lucigenin, and 100 μM NADPH. Photon emission in terms of relative light units was measured in a Glomax luminometer (Promega) every 30 s for 5 min. There was no measurable activity in the absence of NADPH. Superoxide anion production was expressed as relative chemiluminescence (light) units (RLU)/mg protein.

### Luciferase reporter assay

BEAS-2B cells transfected with the luciferase reporter constructs were seeded into 24-well plates (5 × 10^4^/well) and subjected to various treatments when cultures reached 80–90% confluence. Cellular lysates were subjected to a luciferase reporter assay (Promega, Madison, WI) using Glomax luminometer (Promega) as described previously [45]. The results are expressed as relative activity normalized to the luciferase activity in the control cells without treatment.

### ELISA for IL-6 and STAT3

BEAS-2B cells were cultured in DMEM supplemented with 10% FBS containing Cr(VI) (0, 2.5, and 5 μM) for 24 h. Subsequently, the culture media was collected and used to estimate IL-6 level using commercially available IL-6 ELISA kit (BioLegend, Inc. CA, USA) according to the manufacturers' recommendation.

For quantitative analysis of STAT3, Trans^AM^ ELISA kit (Active Motif, Carlsbad, CA) was used following the manufacturer's protocol. For this assay, the nuclear extracts of cell samples from various treatment groups were prepared using the Nuclear Extraction kit (Active Motif) according to the manufacturer's direction. Absorbance was recorded at 450 nm with reference taken at 650 nm. The assay was performed in duplicate and the results are expressed as the percentage absorbance of control group.

### Anchorage-independent colony growth assay for Cr(VI)-induced cell transformation

Soft agar colony formation assay was performed as described previously [45]. BEAS-2B cells or BEAS-2B cells with stable overexpression of catalase or PDCD4 or BEAS-2B cells with stable knockdown of miR-21 were treated with 0.5 μM Cr(VI). The fresh medium was added every 3 days. After 24 weeks, 1 × 10^4^ cells were suspended in 3 mL culture medium containing 0.35% agar and seeded into 6-well plates with 0.5% agar base layer, and maintained in an incubator for 4 weeks. The colonies greater than 0.1 mm in diameter were scored by microscopic examination.

The Cr(VI)-transformed cells from anchorage-independent colonies were selected and grown in DMEM. Passage-matched cells without Cr(VI) treatment were used as control.

### Collagen matrix invasion assay

Collagen matrix invasion assay was performed as described previously [57]. Briefly, BEAS-2B cells (10^5^ cells/Transwell) chronically exposed to Cr(VI) were seeded to the top chamber of invasion chamber and incubated 24 h. Cells in top chamber (non-invaded) were removed, and cells on bottom of filter insert (invaded) were fixed with 3.7% paraformaldehyde and stained with 0.5% crystal violet in 2% ethanol. Membranes were washed and the dye was eluted with 10% acetic acid. Absorbance was measured at 595 nm using a microtiter plate reader (Beckman coulter). The number of invaded cells was presented relative to untreated controls. Experiments was performed in duplicate and repeated 3 times.

### Chromatin immunoprecipitation (ChIP) assay

ChIP analysis was performed using a PierceTM Agarose ChIP Kit (Thermo Scientific, Rockford, IL). Sheared chromatin was diluted and immunoprecipitated with 2 μg of anti-STAT3, anti-TCF4 or control IgG antibody. DNA protein complexes were eluted from the protein A/G agarose beads using a spin column and were reverse cross-linked by incubating with NaCl at 65°C. The relative TCF4 binding to the c-MYC and uPAR promoters or STAT3 binding to miR-21 promoter was analyzed by MyiQ™ Single-Color Real-Time PCR Detection System (Bio-Rad, Hercules, CA) with SYBR Green PCR master mix using following primer sequences, TCF4 binding to c-Myc promoter: (F) GCGCCCATTAATACCCTTCT and (R) TCTCCCTTTCTCTGCTGCTC; TCF4 binding to uPAR promoter: (F) GGAAGCAAAGCAAGGGTTAAG and (R) GCCCTGACTCATGGAGTTGT; STAT3 binding to miR-21 promoter/enhancer (F) CCTCTGAGAAG AGGGGACAA and (R) ACCGCTTCCAGCA AAAGAGT. General PCR amplification also performed in a Mastercycler^®^ thermal cycler (Eppendorf, Foster City, CA).

### Quantitative real-time polymerase chain reaction (qRT-PCR)

Total RNA was extracted using Trizol (Invitrogen), and cDNA was synthesized by using TaqMan^®^ microRNA reverse transcriptase kit (Applied Biosystems, Foster City, CA, USA) according to the manufacturer recommendations. Expression of miR-21-microRNA was determined by the TaqMan miRNA-assay (Applied Biosystems, Foster City, CA, USA), and normalized using the 2^−ΔΔCT^ -method relative to U6-snRNA. All TaqMan-PCR reactions were performed in triplicates using a Bio-Rad's MyiQTM single-color real-time PCR detection system.

### Human tissue samples

Human lung adenocarcinoma tissue (stage IA or IIA) was provided from the Biospecimen and Tissue Procurement Shared Resource Facility of the University of Kentucky Markey Cancer Center. Lung tissues were freshly frozen and fixed with 10% formalin for real-time PCR and immunofluorescence analysis, respectively.

### Western blot analyses

Cells lysates were prepared in ice-cold RIPA buffer (Sigma-Aldrich) containing freshly added protease inhibitor cocktail. The lysate was then centrifuged at 12000 g for 10 min at 4°C and the supernatant (total cell lysate) was collected, aliquoted and stored at −80°C. The protein concentration was determined using Coomassie Protein Assay Reagent (Thermo, Rockford, IL). About 40 μg cellular proteins were separated using 6%–12% SDS-polyacrylamide gel and transferred to nitrocellulose membrane (Bio-Rad, Hercules, CA). Membranes were blocked with 5% fat-free dry milk in 1X Tris-buffered saline (TBS) and incubated with antibodies. Protein bands were detected by incubating with horseradish peroxidase-conjugated antibodies (Kirkegaard and Perry Laboratories, Gaithersburg, MD) and visualized with enhanced chemiluminescence reagent (Perkin Elmer, Boston, MA). Tumor tissues were homogenized with MagNA Lyser Green Beads using MagNA Lyser Instrument (Roche, Indianapolis, IN) and Western blot analysis performed.

### Chronic exposure of animals to Cr(VI) particle

6–8 weeks old, female BALB/c mice were purchased from Jackson Laboratory (Bar Harbor, MN). Endotoxin-free basic zinc chromate particle was crushed using mortar and pestle and then washed with distill water and acetone to generate a particle of 4.7 μm and a purity of 99–100%. Cr(VI) particle was suspended in sterile 0.9% sodium chloride solution at a concentration of 1.2 mg/ml and prepared as previously described [58]. Animals under a light anesthesia (isoflurane) were intranasally exposed to a 50 μl dose of chromate or saline once a week up to 12 weeks. Lungs of animals were isolated and subject to freshly frozen and 10% formalin fixation for real time PCR and immunohistochemistry analysis, respectively.

### Tumorigenesis studies

Athymic nude mice (NU/NU, 6–8 weeks old; Charles River) were housed in a pathogen-free room in the animal facilities at the Chandler Medical Center, University of Kentucky. Cells (2 × 10^6^ cells per mouse) from different treatments were resuspended in serum-free medium with matrigel basement membrane matrix (BD Biosciences) at a 1:1 ratio (total volume = 100 μl) and subcutaneously injected into the flanks of nude mice. Mice were checked daily for tumor appearance, and tumor volume was measured every 3 days for 30 days. Tumor volume was determined by Vernier caliper, following the formula of *A* × *B*^2^ × *0.52*, where A is the longest diameter of tumor and B is the shortest diameter. At the end of the experiment, mice were sacrificed, the tumors excised and snap frozen.

### Immunohistochemical staining

Five-μm thick frozen tumor sections were hydrated in phosphate buffered saline (PBS), and non-specific binding sites were blocked with 10% horse serum in PBS. Staining was performed using Vectastain ABC Kit according to the manufacturer's protocol (Vector Laboratories, Burlingame, CA). Briefly, the sections were incubated with rabbit anti-PDCD4 (1:100) antibody for 2 h at room temperature, washed and then incubated with biotinylated secondary antibody for 45 min followed by incubation with ABC reagent. After washing in PBS, color was developed with DAB solution until the desired staining intensity was achieved. Finally, the sections were counterstained with hematoxylin.

### Immunofluorescence analysis

BEAS-2B cells or BEAS-2B cells chronically exposed to Cr(VI) were grown on coverslips in 6-well plates and treated with Cr(VI). The cells were fixed in 4% paraformaldehyde followed by permeabilization with 0.2% Triton X-100, blocked with 10% horse serum in PBS solution, and incubation with antibodies PDCD4 (1:500), E-cadherin (1:100), β-Catenin (1:100), TCF4 (1:100) in buffer A (1% BSA, 0.1% Triton X-100, 10% horse serum in PBS solution) for 1 h at 37°C. The cells were then incubated with Alexa Fluor 594 goat anti-rabbit or Alexa Fluor 488 goat anti-rabbit secondary antibody and mounted using DAPI. The cells were visualized using digital confocal microscopy (Confocal Fluorescence Imaging Microscope, Leica TCS-SP5) or Olympus BX53 fluorescence microscope.

For the formalin-fixed lung tissue fluorescence immunohistochemical analysis, air-dried tissue sections were rinsed, blocked, and incubated with primary antibody overnight. Second antibody was added for 1 h. 300 μl of the diluted DAPI solution was added and incubated 2–5 min at room temperature. DAPI binds to DNA and is a convenient nuclear counterstain. The sections were mounted with an anti-fade mounting media. Olympus BX53 fluorescence microscope was used for visualization.

### Statistical analysis

Presented values are means ± SD. One-way analysis of variance (ANOVA) was used for statistical analysis, with *p* < 0.05 was considered significantly different.
